# Supplementary material for: Disentangling genetic and environmental risk factors for individual diseases from multiplex comorbidity networks
Source: Sci Rep. 2016 Dec 23;6:39658. doi: 10.1038/srep39658 (PMC5180180; doi:10.1038/srep39658)
Supplement: Supplementary Information [file srep39658-s1.pdf]

Supporting Information: Disentangling genetic and environmental risk factors for individual diseases from multiplex comorbidity networks

Peter Klimek[1], Silke Aichberger[1], and Stefan Thurner[1,2,3,*]

[1]Section for Science of Complex Systems; CeMSIIS, Medical University of Vienna; Spitalgasse 23; A-1090; Austria
[2]Santa Fe Institute; 1399 Hyde Park Road; Santa Fe; NM 87501; USA
[3]IIASA, Schlossplatz 1, A 2361 Laxenburg; Austria
[*]stefan.thurner@meduniwien.ac.at

ABSTRACT

Text S1: Further details on data extraction and MIM-ICD10 mappings

Genetic disease associations are extracted from the Online Mendelian Inheritance in Man (OMIM) dataset, from which we obtained a list of 4,847 associations between disorders (phenotype MIM numbers) and genes[1]. Thereby we included only those phenotype-gene associations for which the molecular basis of the disorder is known (i.e. a phenotype mapping key with value 3 in the OMIM dataset). *Metabolic* disease associations stem from the UniProtKB database, which provides a list of 3,020 proteins that are known to be involved in disorders in humans (given by phenotype MIM numbers)[2]. The REACTOME database cross-references these proteins with pathways in which they occur[3]. Toxicogenomic disease associations are obtained from the Comparative Toxicogenomic Database as a list of 4,925 associations between disorders (phenotype MIM numbers or MeSH ID) and chemicals[4]. Here we only use curated disease-chemical associations, i.e. those for which direct, literature-curated evidence exists.

To obtain mappings from MIM phenotype numbers and MeSH codes to the ICD10 classification we compile three different data sources. In addition to the Human Disease Ontology database[5] and mappings provided from OrphaNet[6], we extracted mappings by crawling a disease index page from Wikipedia (https://en.wikipedia.org/wiki/ICD-10, retrieved 04/30/2015). From these three sources result 85,303 MeSH-ICD10 and 5,498 MIM-ICD10 associations. Aggregated to the three-digit ICD10 level, 90% of the ICD10 codes can be mapped to MeSH codes and 37% to MIM numbers. This lower number of successfully translated MIM numbers is partly due to the fact that not for all diseases a molecular basis is known or even relevant.

While the ICD10 codes are primarily used for billing and clinical purposes, the OMIM classification focuses on descriptive phenotypes of inherited conditions. From this follows the limitation that some very specific OMIM codes might link to highly unspecific ICD10 codes and *vice versa*. For instance, colorectal cancer has one MIM number (114500) but four different ICD10 codes on the three-digit level, C18-C21. These four phenotypes are not only connected among each other; each disease that is genetically linked to colorectal cancer is also linked to all four of these ICD10 codes. Consequently the diagnoses C18-C21 have the highest degrees in the genetic comorbidity network. Similarly, the ICD10 codes for essential hypertension (I10-I13) all map to a single MIM number and have the highest degrees in the toxicogenomic disease network. We therefore adjusted for such biases by re-scaling the relative comorbidity risk r_i^α by the node degree k_i^α.

Text S2: Broad-sense heritability

Heritability is a measure that quantifies how much variation in a phenotypic trait (such as a disease) in a population is due to genetic variation among individuals in the population. More specifically, if σ_G is the genetic variation and σ_P the variation in the population, the broad-sense heritability, H^2, is defined as $H^2 = \frac{\sigma_G}{\sigma_P}$. Sloppily defined, heritability measures the proportion of (disease) risk that is due to the genetic background of an individual, as opposed to environmental factors. However, high values of heritability do not necessarily imply a high disease risk, as it may be relatively easy to prevent certain genetic diseases by certain interventions.

Table 1. Overview of characteristics of the HDMN layers. The numbers of non-isolated nodes, N^{α}, and links, L^{α}, are given for four different layers.

α	N^{α}	L^{α}
phenotypic	358	63903
genetic	285	969
pathway-based	251	3930
toxicogenomic	199	4994

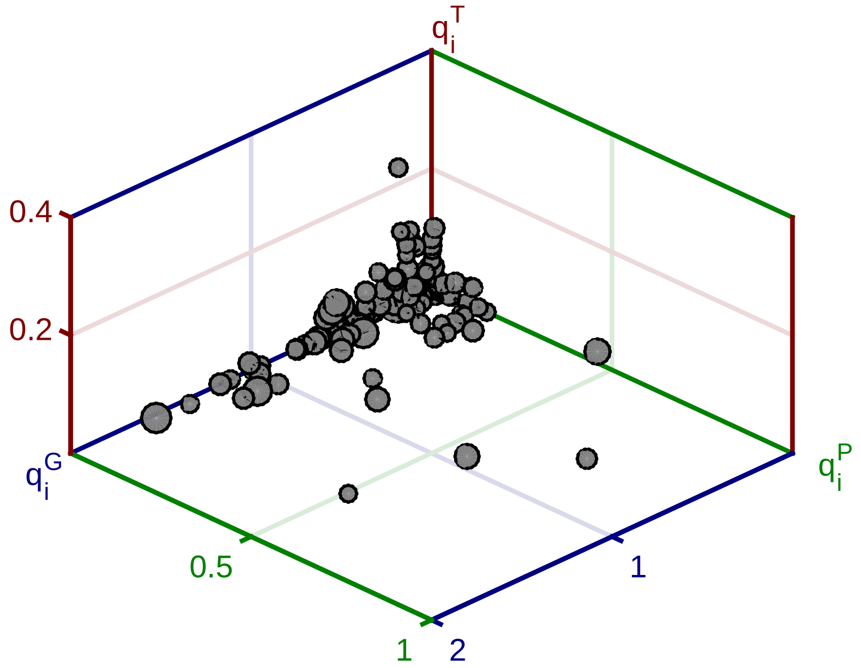

Figure 1. Classification of diseases (circles) according to the dominant contributions to their phenotypic comorbidities for an alternative definition of the re-scaled relative comorbidity risks, q_i^{α}, where two diseases can at the same be comorbid in a genetic of pathway-based / toxicogenomic way. Again, most diseases cluster around one of the axis with a clear dominance of genetic comorbidity risks.

References

1. Online Mendelian Inheritance in Man, OMIM. McKusick-Nathans Institute of Genetic Medicine, Johns Hopkins University (Baltimore, MD).

2. The UniProt Consortium, Activities at the Universal Protein Resource. *Nucleic Acids Research* **42**, D191–8 (2014).

3. Croft, D. *et al.* The reactome pathway knowledgebase. *Nucleic Acids Research* **42**, D472–7 (2014).

4. Davis, A. P. *et al.* The comparative toxicogenomics database's 10th year anniversary: update 2015. *Nucleic Acids Research*, D914–20 (2014).

5. Osborne, J. D. *et al.* Annotating the human genome with disease ontology. *BMC Genomics* **10**(Suppl1), S6 (2009).

6. Aymé, S. & Schmidtke, J., Networking for rare diseases: a necessity for Europe. *Bundesgesundheitsblatt Gesundheitsforschung Gesundheitsschutz* **50**(12), 1477–83 (2007).
